# Supplementary material for: Using Bacillus subtilis as a Host Cell to Express an Antimicrobial Peptide from the Marine Chordate Ciona intestinalis
Source: Mar Drugs. 2021 Feb 12;19(2):111. doi: 10.3390/md19020111 (PMC7918927; doi:10.3390/md19020111)
Supplement: Supplementary file 1 [file marinedrugs-19-00111-s001.pdf]

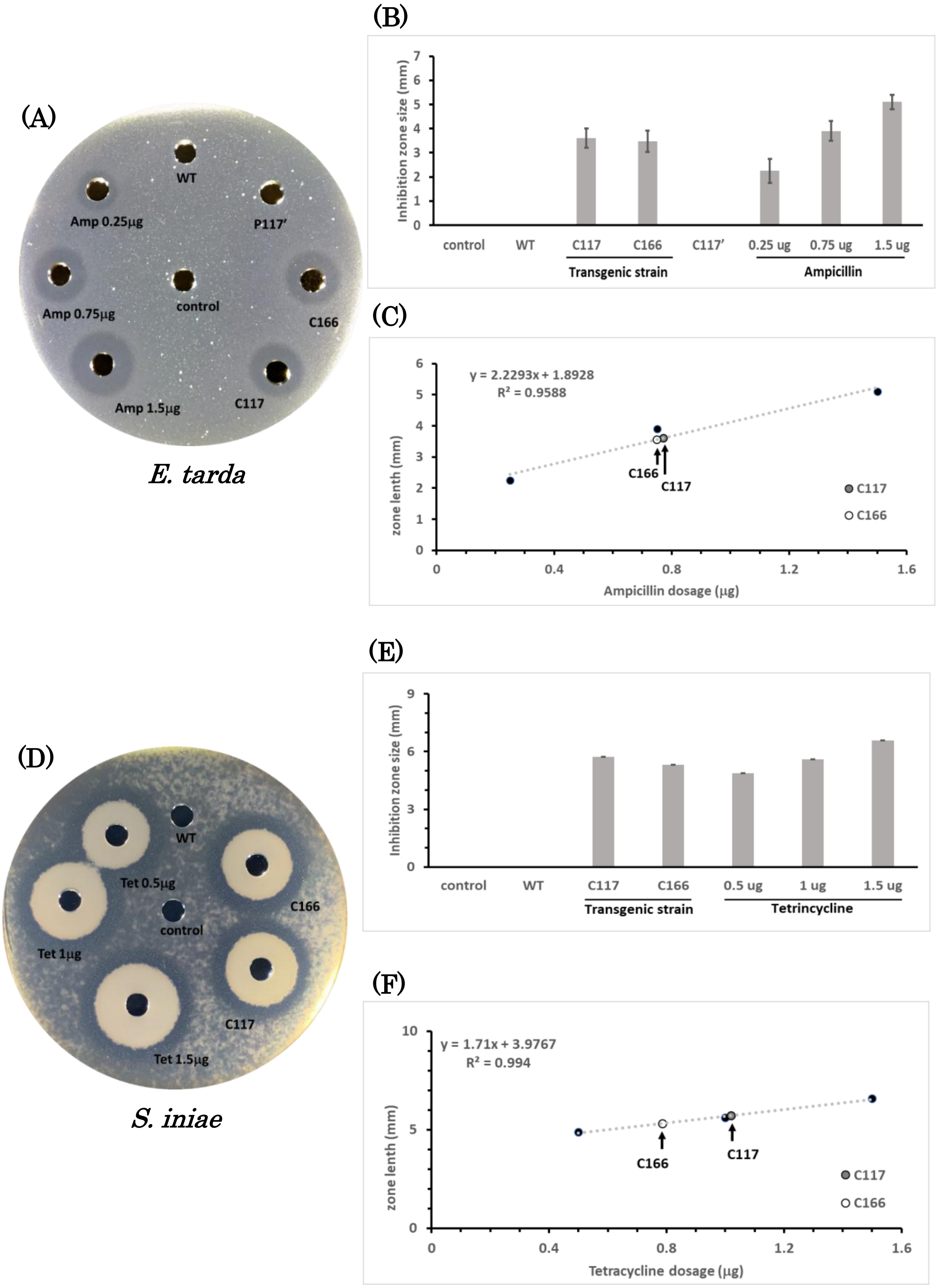

Supplementary Figure S1. Agar well diffusion assay to demonstrate that the extract from transgenic *B. subtilis* strains exhibits bactericidal activity against euryhaline pathogens. (A) Extracts from the transgenic *B. subtilis* C117 and C166 strains were added into the well on agar plate confluent with pathogen *E. tarda.* Ampicillin (Amp) served as a positive control, and three dosages, as indicated, were used to generate a standard curve for calculating the antibacterial potency of the extracts from *B. subtilis.* Distilled water (control) and the extract from non-transformed *B. subtilis* WB800 (WT) served as negative controls. The extract obtained from transgenic

B. subtilis C117 without pepsin treatment (C117’) served as a mock control, while the extract from C117 treated with pepsin (C117) was an experimental group. (B) Each inhibition zone shown around the loading wells was measured and presented as a bar chart (n=6). (C) Three dosages indicated on the plate were used to generate a linear trendline for obtaining a linear regression equation shown on the left. Based on this equation, the equivalent amount of antibiotic potency against *E. tarda* from extracts of transgenic *B. subtilis* C117 and C166 strains, were generated. (D-F) A similar strategy was used to examine *S. iniae*.

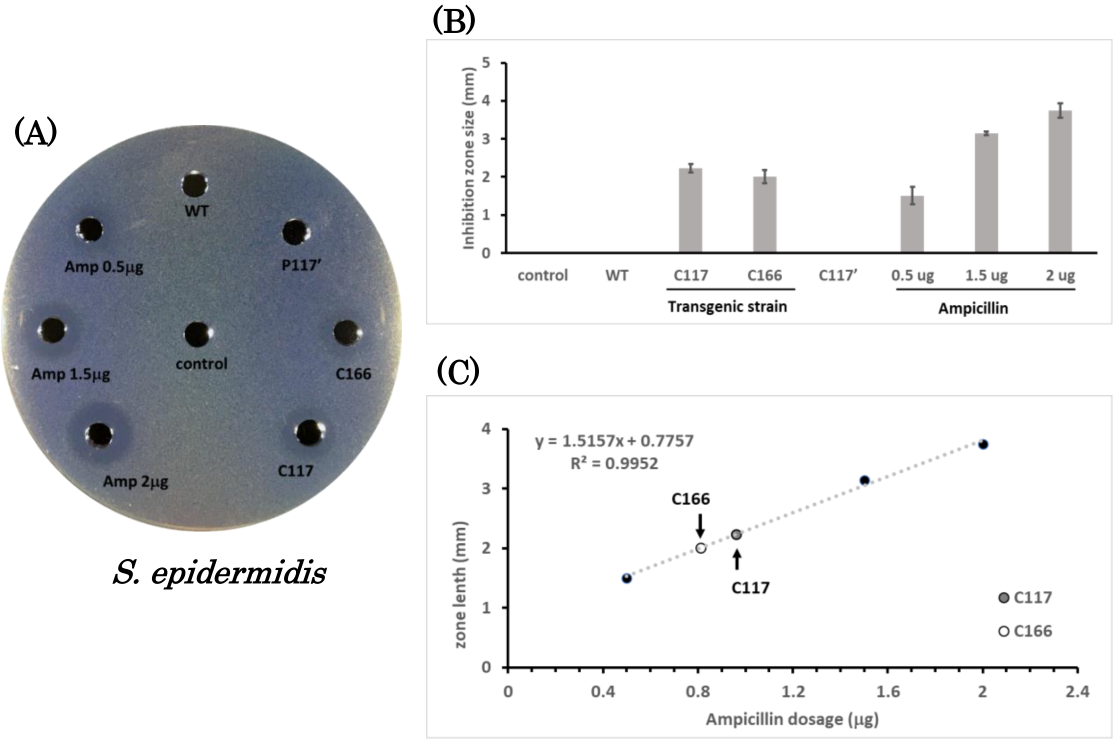

Supplementary Figure S2. Agar well diffusion assay to demonstrate that the extract from transgenic *B. subtilis* strains exhibits bactericidal activity against freshwater pathogen. (A) The extracts from the transgenic *B. subtilis* C117 and C166 strains were added into the well on agar plate confluent with pathogen *S. epidermidis.* Ampicillin (Amp) served as a positive control, and three dosages, as indicated, were used to generate a standard curve for calculating the antibacterial potency of the extracts from *B. subtilis.* Distilled water (control) and the extract from non-transformed *B. subtilis* WB800 (WT) served as negative controls. The extract obtained from transgenic *B. subtilis* C117 without pepsin treatment (C117’) served as a mock control, while the extract from C117 treated with pepsin (C117) was an experimental group. (B) Each inhibition zone shown around the loading wells was measured and presented as a bar chart (n=6). (C) Three dosages indicated on the plate were used to obtain a linear regression equation shown on the left. Based on this equation, the equivalent amount of antibiotic potency against *S. epidermidis* from the extract of transgenic *B. subtilis* C117 and C166 strains, were generated.

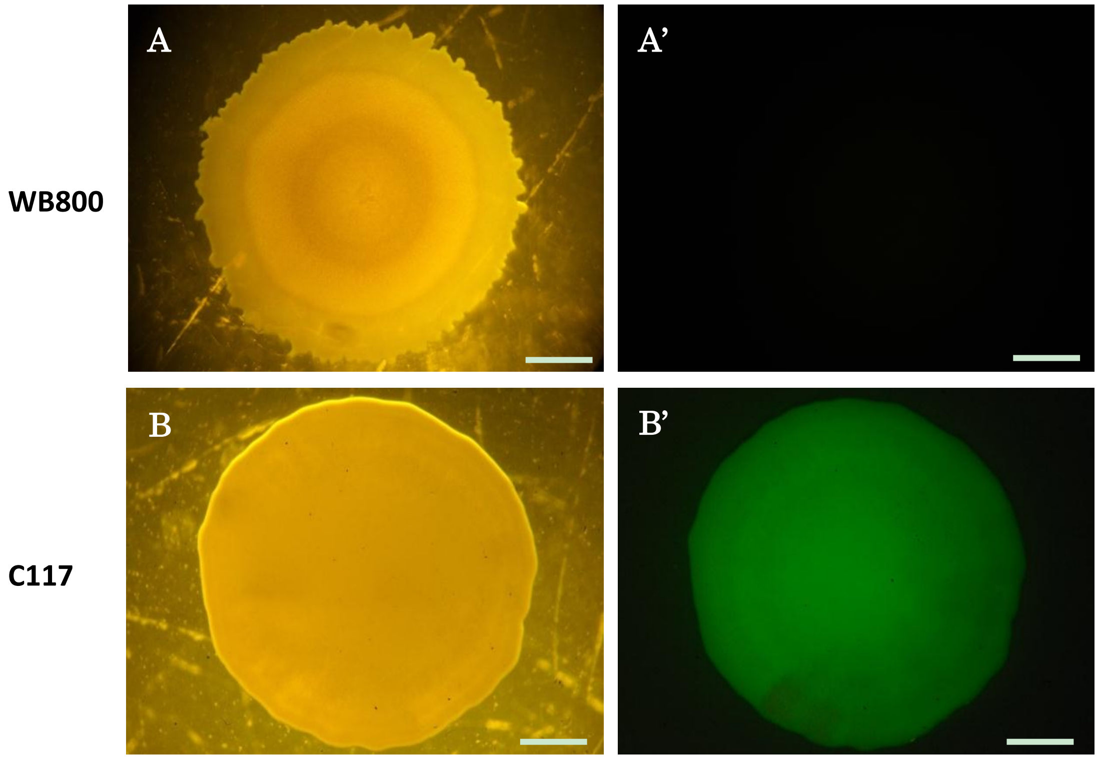

Supplementary Figure S3. The green fluorescence signal is apparent in the recombinant CiMAM-expressing transgenic *B. subtilis* strain. (A-B) The eight-time magnified images under bright field microscope of (A) non-transgenic *B. subtilis* WB800 strain and (B) transgenic *B. subtilis* C117 strain grown on agar plate for seven days. (A′-B′) The eight-time magnified images under fluorescence microscopy equipped with a 470-490 nm excitation filter and a 520-590 nm emission filter. The green fluorescence signal could not be observed in (A′) WB800 strain, while the green fluorescence signal was clearly observed in (B′) C117 strain. Scale bar: 2 mm.
